# Miller–Fisher syndrome after coronary artery bypass surgery

**DOI:** 10.5830/CVJA-2017-033

**Published:** 2017

**Authors:** Aldag Mustafa, Albeyoglu Sebnem, Ciloglu Ufuk, Kutlu Hakan, Ceylan Levent

**Affiliations:** Department of Cardiovascular Surgery, Siyami Ersek Thoracic and Cardiovascular Surgery Training and Research Hospital, Istanbul, Turkey; Department of Cardiovascular Surgery, Siyami Ersek Thoracic and Cardiovascular Surgery Training and Research Hospital, Istanbul, Turkey; Department of Cardiovascular Surgery, Siyami Ersek Thoracic and Cardiovascular Surgery Training and Research Hospital, Istanbul, Turkey; Department of Cardiovascular Surgery, Siyami Ersek Thoracic and Cardiovascular Surgery Training and Research Hospital, Istanbul, Turkey; Department of Cardiovascular Surgery, Siyami Ersek Thoracic and Cardiovascular Surgery Training and Research Hospital, Istanbul, Turkey

**Keywords:** Miller–Fisher syndrome, Guillain–Barre syndrome, coronary artery bypass grafting, cardiopulmonary bypass

## Abstract

Miller–Fisher syndrome (MFS) is an uncommon neurological disorder that is considered a variant of the Guillain–Barre syndrome (GBS). It is clinically defined by a triad of symptoms, namely ataxia, areflexia and ophthalmoplegia. These acute inflammatory polyradiculopathic syndromes can be triggered by viral infections, major surgery, pregnancy or vaccination. While the overall incidence of GBS is 1.2–2.3 per 100 000 per year, MFS is a relatively rare disorder. Only six cases of GBS after cardiac surgery have been reported, and to our knowledge, we describe the first case of MFS after coronary artery bypass surgery. Although cardiac surgery with cardiopulmonary bypass may increase the incidence of MFS and GBS, the pathological mechanism is unclear. Cardiac surgery may be a trigger for the immune-mediated response and may cause devastating complications. It is also important to be alert to de novo autoimmune and unexpected neurological disorders such as MFS after coronary bypass surgery.

Miller–Fisher syndrome (MFS) is an uncommon neurological disorder that is considered a variant of Guillain–Barre syndrome (GBS).^1^ MFS is clinically defined by a triad of symptoms, namely ataxia, areflexia and ophthalmoplegia. Both syndromes usually occur after viral infections, mostly by Campylobacter jenuni, cytomegalovirus, and Epstein–Barr and influenza viruses. These acute inflammatory polyradiculopathic syndromes can also be triggered by major surgery, pregnancy or vaccination.2 While the overall incidence of GBS is 1.2–2.3 per 100 000 persons per year,3 MFS is a relatively rare disorder, accounting for approximately 5% of patients with GBS.^4^

GBS is rare among post-surgical inflammatory neuropathies. Only six cases of GBS after cardiac surgery have been reported,^5^ and to our knowledge, we have described the first case of MFS after coronary artery bypass surgery.

## Case report

A 50-year-old man was admitted to the emergency department with a four-hour history of angina pectoris. Anterior ST-segment elevation myocardial infarction was confirmed and the patient underwent coronary angiography.

Triple-vessel coronary artery disease was demonstrated on catheterisation, requiring urgent coronary bypass surgery. A successful emergency coronary artery bypass procedure (left internal thoracic artery to the left anterior descending artery, and saphenous vein graft to the circumflex and diagonal arteries) was performed using cardiopulmonary bypass (CPB) with 30°C hypothermia.

Alhough the pre- and intra-operative periods remained uneventful, the patient noticed ataxia, left-sided ptosis, weakness and paresthaseia of his legs, which progressed rapidly on the fifth postoperative day. Ataxia was prominent in the lower extremities during standing and walking. There was no history of viral infection, fever or other neurological diseases.

On neurogical examination, unilateral ptosis, gait ataxia and areflexia were noted. After neurology consultation, cranial computerised tomography (CT) revealed nothing unusual. Brain CT and cerebrospinal fluid (CSF) analysis yielded normal results.

CSF viral serology and gram stain culture were negative. Additional laboratory work-up, including tests for connective tissue disorders, anti-thyroid peroxidase and anti-thyroglobulin antibodies were within normal limits. Electromyography and brain magnetic resonance imaging (MRI) were performed and a possible diagnosis of Miller–Fisher syndrome was considered.

Urgent plasmapheresis treatment was planned but within 24 hours the patient had serious dysphagia and rapidly developed dyspnoea. After elective intubation, the patient was transfered to the neurology intensive care unit. Treatment with plasmapheres and intravenous immunoglobulin (0.4 g/kg/daily) was started immediately. Although inotropic support and medications were given to the patient, cardiopulmonary arrest occured on the ninth postoperative day and he died inauspiciously.

## Discussion

Although GBS has occasionally been reported after cardiac surgery, there is no case report in the literature of MFS after coronary artery bypass surgery.^5^ To our knowledge, we describe the first case of MFS after coronary bypass surgery.

Although cardiac surgery with CPB may increase the incidence of MFS and GBS, the pathological mechanism is unclear.^6^ A humoral immune response with deposition of complements and immunoglobulins and a cellular response of infiltrating macrophages and T cells are the most common hypotheses on the underlying mechanism of these syndromes.7 After cardiac surgery, several factors may be triggered to initiate an inflammatory response. These include cardiopulmonary bypass (extracorporeal circulation), ischaemia and reperfusion injury.

When patients present with rapidly progressive paralysis, a diagnosis of GBS and variants such as MFS need to be made as soon as possible. The diagnosis is largely based on clinical patterns because radiological and diagnostic markers are not available for most variants of the syndrome. MFS is a clinical diagnosis, but additional investigations may be helpful or even necessary for confirmation.

Examination of CSF is important, especially to exclude other causes of weakness associated with an increase in CSF cell count.8 Nerve conduction studies may help support the diagnosis, to discriminate between axonal and demyelinating subtypes, but nerve conduction abnormalities are most pronounced two weeks after the start of weakness.^9^

Severe, generalised manifestations of GBS and MFS with respiratory failure affect 20 to 30% of cases.^10^ Treatment with intravenous immunoglobulin or plasma exchange is the optimal management approach, alongside supportive care. Intravenous administration of high-dose immunoglobulin was found to be as effective as plasma exchange.^11^

## Conclusion

MFS, which is a variant of GBS, is a rare but severe neurological complication after cardiac surgery. Cardiac surgery may be a trigger for immune-mediated responses and may cause devastating complications. It is important to be alert to de novo autoimmune and unexpected neurological disorders such as MFS after coronary bypass surgery.
